# Prognostic Impact of Metabolic Syndrome and Steatotic Liver Disease in Hepatocellular Carcinoma Using Machine Learning Techniques

**DOI:** 10.3390/metabo14060305

**Published:** 2024-05-27

**Authors:** Sergio Gil-Rojas, Miguel Suárez, Pablo Martínez-Blanco, Ana M. Torres, Natalia Martínez-García, Pilar Blasco, Miguel Torralba, Jorge Mateo

**Affiliations:** 1 Gastroenterology Department, Virgen de la Luz Hospital, 16002 Cuenca, Spain; 2 Medical Analysis Expert Group, Instituto de Investigación Sanitaria de Castilla-La Mancha (IDISCAM), 45071 Toledo, Spain; 3 Medical Analysis Expert Group, Institute of Technology, Universidad de Castilla-La Mancha, 16071 Cuenca, Spain; 4 Internal Medicine Unit, University Hospital of Guadalajara, 19002 Guadalajara, Spain; 5 Department of Pharmacy, General University Hospital, 46014 Valencia, Spain; 6 Faculty of Medicine, Universidad de Alcalá de Henares, 28801 Alcalá de Henares, Spain; 7 Translational Research Group in Cellular Immunology (GITIC), Instituto de Investigación Sanitaria de Castilla-La Mancha (IDISCAM), 45071 Toledo, Spain

**Keywords:** metabolic dysfunction-associated steatotic liver disease, hepatocellular carcinoma, MASLD-related HCC, liver cirrhosis, mortality, machine learning, extreme gradient boosting

## Abstract

Metabolic dysfunction-associated steatotic liver disease (MASLD) currently represents the predominant cause of chronic liver disease and is closely linked to a significant increase in the risk of hepatocellular carcinoma (HCC), even in the absence of liver cirrhosis. In this retrospective multicenter study, machine learning (ML) methods were employed to investigate the relationship between metabolic profile and prognosis at diagnosis in a total of 219 HCC patients. The eXtreme Gradient Boosting (XGB) method demonstrated superiority in identifying mortality predictors in our patients. Etiology was the most determining prognostic factor followed by Barcelona Clinic Liver Cancer (BCLC) and Eastern Cooperative Oncology Group (ECOG) classifications. Variables related to the development of hepatic steatosis and metabolic syndrome, such as elevated levels of alkaline phosphatase (ALP), uric acid, obesity, alcohol consumption, and high blood pressure (HBP), had a significant impact on mortality prediction. This study underscores the importance of metabolic syndrome as a determining factor in the progression of HCC secondary to MASLD. The use of ML techniques provides an effective tool to improve risk stratification and individualized therapeutic management in these patients.

## 1. Introduction

Hepatocellular carcinoma (HCC) is the most common primary liver tumor, positioned as the sixth most commonly diagnosed cancer worldwide [1]. The survival rate barely reaches 20% at 5 years, making it one of the deadliest tumors [2,3]. Despite universal vaccination policies in newborns against hepatitis B virus (HBV) and new antiviral treatments for hepatitis C virus (HCV), an increase in their incidence is anticipated in the coming years [4]. This is due to increased alcohol consumption and the rise of metabolic dysfunction-associated steatotic liver disease (MASLD) [5,6].

Most of these tumors occur in cirrhotic livers, with limited functional hepatic reserve, which complicates adequate therapeutic management [7]. Because of the deteriorated baseline condition in many patients, the presence of other comorbidities related to the development of metabolic syndrome is common. Additionally, in this group of patients, the presence of eating disorders is often observed, and the combination with even non-harmful alcohol consumption may promote the development of hepatic steatosis [8]. It is known that the metabolic profile of these patients, in addition to being associated with an increased risk of cardiovascular disease and promoting the development of other conditions such as polycystic ovary syndrome (PCOS), chronic kidney disease (CKD), and osteoporosis, increases the risk of various tumors, including HCC [9,10].

Despite the development in recent years of dynamic imaging techniques, which have allowed for the diagnosis of HCC in cirrhotic livers, liver biopsy is still necessary in non-cirrhotic livers or in cirrhotic livers that do not exhibit a typical radiological behavior according to the Liver Imaging Reporting and Data System (LI-RADS) criteria [11]. There are different prognostic and therapeutic scales, none of which are universally valid [12]. This is because there are different risk factors related to the development of these tumors in various geographic regions, which could influence the disease progression [13,14]. The Barcelona Clinic Liver Cancer (BCLC) classification is the most commonly used in our setting because, unlike other classifications, such as tumor node metastasis (TNM), which only take into account the tumor’s own characteristics, it considers individual features such as the degree of functional hepatic reserve and the patient’s baseline status [15].

At present, only about 30% of patients diagnosed with HCC can benefit from curative-intent treatment [16,17]. Due to the poor prognosis of these tumors, it is essential to implement adequate screening programs in high-risk groups for early detection. Performing biannual ultrasound surveillance is advisable for all cirrhotic patients with the exception of those classified as Child–Pugh class C who are not eligible for liver transplantation. Additionally, it is advisable for patients with advanced fibrosis F3-F4 and non-cirrhotic patients with HBV infection [18]. At present, there is no molecule or biomarker available capable of predicting the course of the disease or facilitating its diagnosis in early stages for inclusion in the screening program. Although the determination of alpha-fetoprotein (AFP) is widely used due to its easy availability, it does not provide benefits for screening, while its true utility in the prognosis of these patients is still debated [19]. Therefore, it is crucial to focus on researching other factors and signaling pathways related to the pathogenesis of these tumors, which could impact patient survival by favoring their detection in early stages. Exploring these approaches could lead to the development of more effective screening strategies and enable the implementation of personalized treatments for better patient outcomes [20].

Due to the significant increase in metabolic syndrome among the population in recent years and its direct relationship with the development of these tumors, the following study is proposed to evaluate the influence of a worse metabolic profile on the survival of these patients. Identifying these factors will enable the implementation of specific interventions designed to improve outcomes in terms of life expectancy [21]. For this purpose, machine learning (ML) techniques will be employed. The application of these learning methods enables the efficient handling of large amounts of data, allowing for reliable predictions of outcomes compared to conventional statistics [22,23]. The eXtreme Gradient Boosting (XGB) algorithm was chosen as the method for developing the learning model, and it was compared with other systems commonly used in the scientific literature [24]. XGB was selected for its fast execution, its ability to adapt to large volumes of data, and its accuracy in results obtained to date in various areas of clinical practice [19,25].

## 2. Materials and Methods

### 2.1. Study Design and Population

A retrospective multicenter cohort study was conducted at the Virgen de la Luz Hospital in Cuenca and the University Hospital of Guadalajara. All patients over 18 years of age diagnosed with HCC using imaging techniques or histological studies from 2008 to 2023 were included, totaling 219 cases. Patients with a previous diagnosis at another healthcare facility without knowledge of prognostic variables at the time of diagnosis were excluded from the study. The study was approved by the Ethics Committee of the University Hospital of Guadalajara, and obtaining informed consent from patients was not considered necessary.

### 2.2. Study Data

The study included variables commonly associated with metabolic syndrome and the progression of MASLD [26]. The demographic variables included gender and age at the time of HCC diagnosis. Age was obtained by calculating the difference between the diagnosis date and the date of birth. The censoring date for each patient in the study corresponded to the date of death for deceased patients and the date of the last medical visit for those who remained alive. Variables related to toxic habits were analyzed, such as harmful alcohol consumption, defined as intake of more than 30 g per day in males and 20 g per day in females [27]; within the smoker category, patients with active smoking or former smokers were included, compared to those who had never smoked.

The main etiologies related to the development of HCC were collected, including HCV, HBV, alcohol, MASLD, hemochromatosis, autoimmune hepatitis, primary biliary cholangitis, Wilson’s disease, porphyrias, aflatoxins, and alpha-1 antitrypsin deficiency. Variables related to the diagnostic criteria of metabolic syndrome in adults were analyzed. High blood pressure (HBP) was diagnosed based on medical history with blood pressure readings ≥ 130/85 mmHg and/or the use of antihypertensive medications; body mass index (BMI), calculated using the formula weight (kg)/height^2^ (m^2^), was used to define obesity (≥30 kg/m^2^) [28,29].

The presence of liver cirrhosis was defined according to clinical or radiological criteria [30]; the diagnosis of HCC was achieved through invasive procedures or radiological criteria for those cirrhotic patients with typical behavior. It was distinguished between cirrhotic patients included in screening programs with biannual ultrasound and those who were not included in close surveillance programs. The degree of functional hepatic reserve was assessed using the most representative scales in our setting, such as the Child–Pugh classification and the model for end-stage liver disease (MELD). The presence of clinically significant portal hypertension was defined as an increase in the hepatic venous portal pressure gradient of more than 10 mmHg, the appearance of ascites, or the presence of esophagogastric varices [31]. The overall health status of the patient was defined according to the Eastern Cooperative Oncology Group (ECOG) classification [32]. Within the tumor’s own characteristics, the number of space-occupying lesions (SOLs), the maximum diameter of SOL in cm, and the presence of portal vein thrombosis, pathological lymph nodes, or metastases at the time of diagnosis were recorded. The two most commonly used prognostic and therapeutic scales in our setting, BCLC and TNM, were employed [33].

Laboratory data included serum glucose (mg/dL), glycosylated hemoglobin (HbA1c %), total cholesterol (TC) (mg/dL), high-density lipoprotein cholesterol (HDL-C) (mg/dL), low-density lipoprotein cholesterol (LDL-C) (mg/dL), triglycerides (TG) (mg/dL), vitamin D (ng/mL), total calcium (mg/dL), serum iron (mcg/dL), ferritin (ng/mL), transferrin (mg/dL), transferrin saturation index (TSI %), fibrinogen (mg/dL), uric acid (mg/dL), gamma-glutamyl transpeptidase (GGT) (U/L), and alkaline phosphatase (ALP) (U/L). Within the analytical variables, the ratio between total cholesterol and HDL cholesterol (TC/HDL ratio) was used [34]. In addition, non-invasive fibrosis indices (NITs) were used: fibrosis-4 index (FIB-4) and aspartate aminotransferase/platelet ratio index (APRI) [35].

### 2.3. Development Model

For the statistical analysis, variables were collected in an anonymized database. The analysis focused on prognostic factors at the diagnosis of HCC, with special attention to variables related to metabolic syndrome and its association with the development of MASLD, using ML methods.

The proposed XGB algorithm was selected to develop the predictive model due to its adaptability, execution speed, and compatibility with parallel computing, distinguishing it from other ML methods. XGB also offers the possibility of second-order regularization, which helps to avoid overfitting, a common problem in ML, by improving the model’s generalization [36,37]. Therefore, this algorithm demonstrates high precision and efficiency compared to other data analysis methods. ML and statistical tools from MATLAB (The MathWorks, Natick, MA, USA; MATLAB 2023a) were used to design the models, and the proposed XGB method was compared with other algorithms such as Support Vector Machine (SVM) [38], Decision Tree (DT) [39], Gaussian Naïve Bayes (GNB) [40], and K-Nearest Neighbors (KNN) [41].

**Figure 1:**
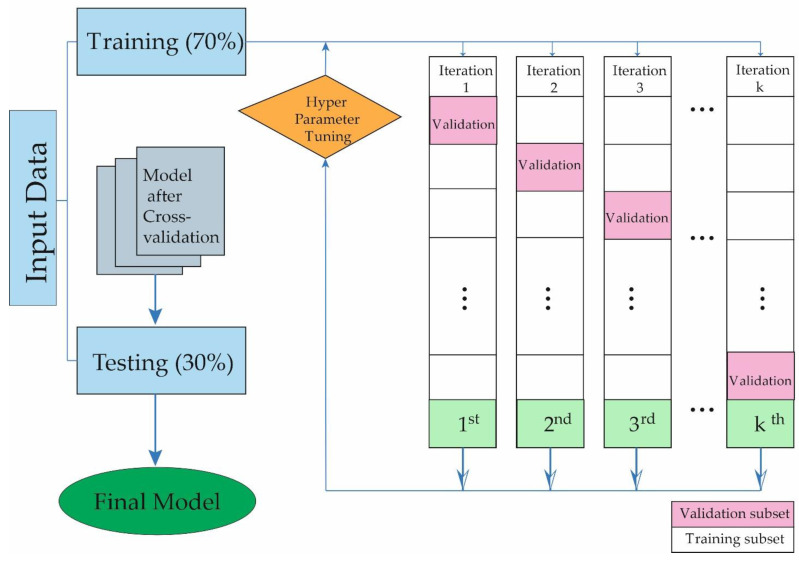
Iterative development process of the machine learning predictive model demonstrating classification performance across training and testing phases.

For the purpose of enhancing algorithm performance, several hyperparameters were fine-tuned during the training phase, using Bayesian techniques to determine optimal values. The Bayesian optimization algorithm, based on sequential models, reduces the need for validation testing by leveraging the results of previous iterations to focus efforts on the most promising hyperparameters. This strategy led to a reduction in the number of model tests and significantly improved the performance of the developed models. To prevent overfitting during the ML process, a k-fold cross-validation technique was employed [42]. Figure 1 depicts a random allocation of 70% of patients for the training phase versus 30% for testing in each iteration. This approach avoided the simultaneous use of patients in both phases to optimize the capture of different aspects of data by the models and reduce overfitting [43]. The preference for XGB stems from its outstanding advantages, positioning it as a superior option in terms of accuracy and flexibility. Unlike GNB, XGB excels in managing irrelevant features, enhancing the robustness and predictive effectiveness of the model. Compared to SVM, XGB demonstrates a unique ability to handle complex and high-dimensional datasets while maintaining notable computational efficiency. Its ensemble approach reduces the risk of overfitting and produces more generalized and predictive models. Additionally, XGB is more resistant to data noise and variability compared to KNN, ensuring greater reliability in decision making. In summary, XGB offers solid and accurate predictive models, especially in complex environments with large volumes of data, improving model generalization and ensuring reliable results.

## 3. Results

In this retrospective study based on records from two hospitals in Castilla-La Mancha (Cuenca and Guadalajara), 219 patients diagnosed with HCC between 2008 and 2023 were identified through histological analysis or radiological study.

**Figure 2:**
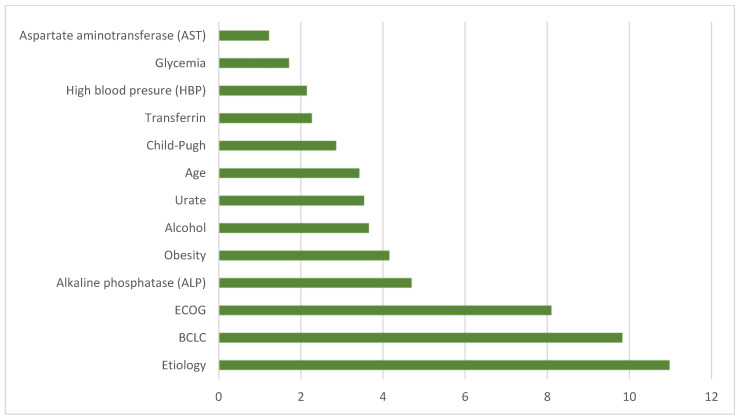
Representation of the contribution of the most influential variables in the predictive model of machine learning.

Figure 2 depicts the relevance of variables in creating a predictive model using ML techniques. It was found that etiology was the most determining factor, followed by BCLC and ECOG classifications. Elevated levels of ALP were identified as an independent predictor of mortality, while some diagnostic criteria for metabolic syndrome such as obesity and HBP were also associated with a worse prognosis, surpassing serum glucose levels in predicting mortality. Alcohol consumption and elevated serum uric acid levels were also shown as independent predictors of mortality. Other prognostic factors included age at diagnosis, Child–Pugh classification, and serum transferrin levels. On the other hand, it was observed that AST levels have less relevance, indicating a lack of significant correlation between elevated levels and patient survival.

Table 1 and Table 2 display the results obtained for various metrics analyzed in the developed models, which include the proposed XGB system along with SVM, DT, GNB, and KNN methods. On one hand, metrics such as balanced accuracy, specificity, sensitivity, and precision were examined. On the other hand, to assess the performance of the methods, metrics such as area under the curve (AUC), Matthews correlation coefficient (MCC), F1 score, Kappa score, and dependent Youden index (DYI) were utilized. As evident from Table 1, XGB exhibits values exceeding 93% for balanced accuracy, sensitivity, and specificity, and very close to this value for precision. This entails a substantial difference compared to the closest method, KNN, with differences of around 6% for these metrics. The discrepancies are even more notable for the rest of the algorithms, particularly with GNB. In this case, the differences are approximately 13%. When analyzing the results of commonly used methods in the scientific literature for validation, such as the dependent Youden index (DYI) and Kappa score, a discrepancy of around 6% is observed between XGB and KNN in favor of the suggested model.

**Figure 3:**
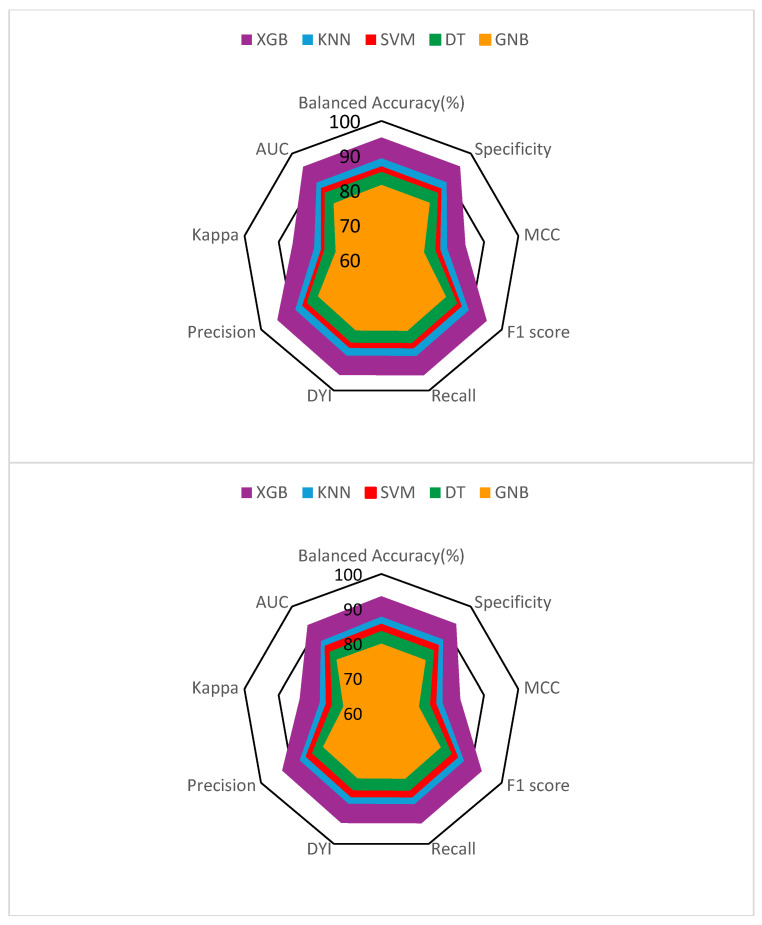
Radar chart illustrating the training phase (**top**) and validation (**bottom**), aimed at establishing the relevance of various prognostic factors in hepatocellular carcinoma within the predictive model. The following abbreviations are used: XGB (eXtreme Gradient Boosting), KNN (K-Nearest Neighbors), SVM (Support Vector Machine), DT (Decision Tree), GNB (Gaussian Naive Bayes).

Conversely, to comprehensively illustrate all these data, a radar chart was prepared. This chart displays the data corresponding to the training phase (located at the top of Figure 3) and the data from the testing phase (located at the bottom of Figure 3). It can be observed that the XGB algorithm exhibits similar data in both phases. This suggests that there is no overfitting, meaning that the resulting model generates good predictive capability with generalization ability. The GNB method yielded the worst results, as it shows a smaller area in this representation, implying lower reliability for the study’s objective.

**Figure 4:**
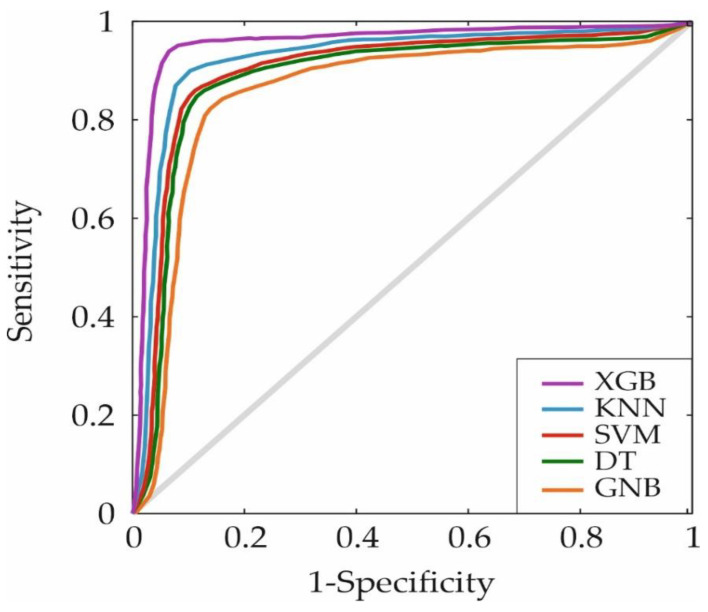
Representation of ROC curves for the five machine learning algorithms. ROC: Receiver Operating Characteristic curve, XGB: eXtreme Gradient Boosting, KNN: K-Nearest Neighbors, SVM: Support Vector Machine, DT: Decision Tree, GNB: Gaussian Naive Bayes.

Finally, Figure 4 illustrates the Receiver Operating Characteristic (ROC) curve, which compares the performance of the XGB system with other algorithms used. This curve represents sensitivity versus specificity for different threshold values. As observed in Figure 4, the XGB method exhibits a broader area under the curve, positioning it as the best algorithm for the study’s purpose. Specific AUC values are detailed in Table 1, where XGB reaches a value of 0.93, followed by KNN with 0.87. This higher AUC implies that it is the optimal method for predicting mortality risk in patients diagnosed with HCC, as well as determining the most relevant variables affecting their mortality.

## 4. Discussion

Recently, in the 2023 Delphi consensus statement, the terms non-alcoholic fatty liver disease (NAFLD) and metabolic dysfunction-associated fatty liver disease (MAFLD) have been replaced by the term MASLD, with the endorsement of leading scientific societies in this field [44]. With this change in nomenclature, the aim is to improve patient identification, promote greater awareness of their condition, while eliminating terms that may have stigmatizing connotations such as ‘non-alcoholic’ or ‘fatty’ [26,45].

MASLD affects more than 30% of the adult population worldwide [46]. Although there are geographical differences, the increase in its prevalence in recent years has made it currently the leading cause of chronic liver disease [47]. In order to establish its diagnosis, demonstrating the presence of steatosis via imaging tests or liver biopsy is essential, along with the presentation of at least one component of the metabolic syndrome in an individual without excessive alcohol consumption. This consumption is defined by an intake of less than 30 g per day in males and 20 g per day in females [48,49]. Among the criteria for metabolic syndrome in adults, highlights include a BMI ≥ 25 kg/m^2^, fasting glucose levels ≥ 100 mg/dL, Hb A1c ≥ 5.7%, HBP ≥ 130/85 mmHg, TG ≥ 150 mg/dL, and serum HDL-C ≤ 40 mg/dL in men and ≤50 mg/dL in women [48]. It is often challenging to distinguish between patients with primary MASLD and those with increased alcohol consumption before or after the diagnosis of MASLD. This consumption contributes to the accelerated progression of liver disease. Therefore, it is proposed that the term Metabolic and Alcohol-Associated Liver Disease (MetALD) be reserved for those patients with MASLD who exhibit higher alcohol consumption, defined as 140–350 g per week in women and 210–420 g per week in men. Patients with demonstrated hepatic steatosis without a known cause, once harmful alcohol consumption is excluded and they do not meet any criteria for metabolic syndrome, are classified under the concept of cryptogenic fatty liver disease [44,50]. Until a few years ago, many patients with liver cirrhosis secondary to MASLD were misclassified as having idiopathic cirrhosis. Hence, it is crucial to have a deeper understanding of the disease and better classification of our patients to facilitate the adoption of increasingly personalized treatments [26].

The pathophysiological mechanisms of the disease, still partially unknown, seem to depend on the interaction between metabolic, inflammatory, and hepatic factors. In MASLD, there is an imbalance between lipid synthesis and elimination, leading to their accumulation in the hepatic parenchyma [51]. Insulin resistance plays a fundamental role in promoting the uptake of fatty acids by the liver, which exceeds its capacity for oxidation and elimination in the form of lipoproteins. As a result of increased fatty acids, there is an increase in intracellular triglyceride synthesis in hepatocytes, which promotes the activation of mechanisms that generate oxidative stress, mitochondrial dysfunction, and processes of programmed cell death, such as ferroptosis [52,53]. With disease progression, there is a parallel increase in the production of proinflammatory cytokines, such as tumor necrosis factor-alpha (TNF-α), interleukin-6 (IL-6), interleukin-32 (IL-32), or transforming growth factor-beta (TGF-β), which exceed the immune system’s control capacity through regulatory T cells [54,55]. This favors the onset of steatohepatitis, which constitutes one of the main pathways for progression to fibrosis and the development of liver cirrhosis [56]. There are numerous molecules and signaling pathways under investigation that could play a prominent role in detecting the disease at early stages and developing new therapeutic targets [57,58]. The study by Syamprasad et al. demonstrates the involvement of AKR1B1 in hepatic lipid accumulation by catalyzing the reduction of aldehyde groups, promoting lipogenesis and hepatic inflammation. Furthermore, its overexpression is associated with HCC progression, contributing to the inhibition of apoptosis, cell proliferation, and angiogenesis [59]. According to the studies by Kurokawa et al. and Takahashi et al., changes in the methylation and transcription of certain genes influence the development of fibrosis and HCC [60,61]. However, other studies such as the one conducted by Kakehashi et al. have focused on the role of mTOR signaling in hepatocarcinogenesis [62]. Recently, the study led by Y. Shi et al. has demonstrated the pivotal role of CLSPN in regulating various signaling pathways associated with cell proliferation and apoptosis. This critical component of the S-phase checkpoint in DNA replication plays a fundamental role in the pathogenesis of HCC by driving cellular activation through signaling pathways such as Wnt/β-catenin. Therefore, the overexpression of CLSPN serves as a suitable prognostic factor in numerous tumors, including HCC, which could facilitate the development of new personalized therapeutic targets for these patients [63].

The progression of this disease is strongly associated with the development of liver fibrosis. The presence of fibrosis is associated with an increase in both overall mortality and disease-related mortality in patients with advanced fibrosis F3-F4 [64,65]. The progression of fibrosis is influenced by multiple factors such as the presence and severity of concomitant diseases (arterial hypertension, diabetes mellitus, dyslipidemia, obesity, etc.) and certain lifestyle factors like a diet rich in saturated fats and refined carbohydrates, lack of physical activity, or alcohol consumption [49]. Additionally, there is a genetic predisposition influenced by environmental exposure to endocrine-disrupting chemicals (EDCs), which could help explain the differences found among different geographical areas [66,67]. On the contrary, coffee consumption may be associated with a lower risk of developing MASLD and hepatic fibrosis [68].

An estimated 10% of patients with MASLD are projected to experience complications associated with chronic liver disease throughout their lifetime, with the emergence of HCC being the most severe [69]. Most of these tumors arise in cirrhotic livers, with an estimated annual risk of developing HCC in patients with cirrhosis secondary to MASLD being approximately 2%. Additionally, these patients are at risk of developing HCC in the absence of liver cirrhosis, which would necessitate a biopsy to establish the diagnosis [5,70]. It is anticipated that in the coming years, MASLD will surpass the main causes historically associated with the development of these tumors, owing to the rise in obesity, the implementation of HBV vaccination strategies in newborns, and the introduction of new treatments for HCV [8,19]. Although only around 1% of MASLD patients will specifically die from liver-related causes, its high prevalence should prompt us to consider this disease. Considering a current Spanish population of approximately 48,593,000 inhabitants and considering a MASLD prevalence in Western Europe of around 32%, it is estimated that more than 15,500,000 people are affected by MASLD in our country, of whom more than 155,000 patients will die from liver-related causes in the coming years [47].

In the conducted study, etiology was the most important variable in predicting mortality at diagnosis in these tumors, followed by BCLC and ECOG classifications. Among the various causes of HCC, patients with a poorer metabolic profile associated with the development of MASLD had a worse prognosis. The BCLC classification, commonly used in our setting, allows for appropriate therapeutic management at each of the diagnostic stages of the disease. This classification incorporates tumor-specific characteristics, the degree of hepatic functional reserve through the Child–Pugh classification and considers the individual baseline status of patients through the ECOG classification. Elevated serum levels of FA showed an inversely proportional correlation with prognosis in these tumors, likely related to obstruction of intrahepatic bile ducts due to replacement of healthy hepatic parenchyma by fibrotic tracts during cirrhosis development, affecting patient survival [71]. The next factor associated with a poorer prognosis is obesity, defined by a BMI ≥ 30 kg/m^2^, which, along with HBP, represents some of the criteria for establishing the diagnosis of metabolic syndrome. Obesity initiates a pro-inflammatory state in the body that promotes the development of hepatic fibrosis, increasing the risk of HCC [72]. Additionally, the sensitivity of screening radiological tests, such as ultrasound, may be reduced in obese patients, making it difficult to detect these tumors at early stages. This circumstance, coupled with the absence of clinical manifestations until we reach advanced stages of MASLD and the possibility of developing HCC in the absence of hepatic cirrhosis, could hinder the application of curative treatments [70]. Type 2 diabetes mellitus (T2DM) has been identified as an independent risk factor for the development of advanced hepatic fibrosis. Therefore, determining through non-invasive scores such as FIB-4 every 1–2 years in those patients with T2DM or who present at least two criteria of metabolic syndrome could facilitate their detection in early stages [8,73].

Alcohol consumption in patients with MASLD, although lower compared to those with pure alcoholic etiology for HCC, is often common. Alcohol intake, regardless of quantity, promotes the development of hepatic steatosis, increases serum urate levels, and elevates blood pressure. On the other hand, alcohol is one of the etiologies of HCC most strongly associated with the development of cirrhosis, which would result in a poorer prognosis for these patients [69,74]. Other factors associated with lower survival include higher scores on the Child–Pugh classification and age, which would hinder the application of curative treatments. Likewise, elevated levels of serum transferrin, as a response to iron overload, have been associated with an unfavorable prognosis by promoting the induction of ferroptosis [75]. However, in the study conducted, high levels of AST are not associated with a worse prognosis, as there is a less pronounced elevation in patients with established cirrhosis, since it is an enzyme primarily synthesized in the liver [76]. Due to the mostly unknown underlying pathophysiological mechanisms of MASLD development, the limitations in including these patients in screening programs until advanced stages of the disease are reached, and the enormous heterogeneity observed in this population, there is a need for the development and implementation of new prognostic factors. This would allow for more precise risk stratification and facilitate the implementation of more personalized therapeutic approaches.

Research was conducted on a largely unexplored area, which is the utilization of ML methods in developing a predictive model to assess the association of metabolic syndrome with hepatic steatosis in the prognosis of patients with HCC. While most studies focus on analyzing pathophysiological mechanisms or molecular markers, a literature search uncovered a study led by Lee et al. [77], which focused on favorable treatment response factors in HCC patients using ML methods. In the conducted study, the XGB method was chosen due to its advantages in versatility, robustness, and accuracy. XGB demonstrated a remarkable ability to handle complex data, achieving values of 93% in most analyzed metrics, with notable efficiency while minimizing the risk of overfitting. Compared to other algorithms, XGB stands out for its capacity to discriminate irrelevant data by reducing noise and enables obtaining reliable results by enhancing the model’s generalization capability when compared with other algorithms. This enables the development of an effective tool to uncover the primary factors affecting the prognosis of patients with HCC. Such a tool assists in clinical decision making by healthcare professionals, thereby contributing to improving patient well-being.

## 5. Conclusions

In conclusion, patients with a poorer metabolic profile who have associated hepatic steatosis have a less favorable prognosis at the diagnosis of HCC. In addition to the most studied prognostic factors to date in these types of tumors, such as BCLC or ECOG classifications, there are other emerging factors related to metabolic syndrome such as obesity, HBP, and elevated levels of FA and urate that may be useful in predicting mortality in this patient group. Even mild alcohol consumption in these patients shows a negative impact in terms of survival.

The proposed XGB algorithm has proven to be an efficient diagnostic tool in recognizing the main predictive factors of mortality in patients with MASLD-related HCC. The most outstanding results were achieved by the XGB method according to the evaluated metrics, with no overfitting. This quality of model generalization makes it a useful resource in everyday clinical practice.

Further studies are needed to identify new prognostic factors, as well as to review screening indications in this patient group, allowing for the detection of these tumors at early stages and providing tailored management to the individual needs of each patient.

## Figures and Tables

**Table 1:** Summary of average results of accuracy, specificity, recall, precision, and AUC obtained from different machine learning models in the study. XGB: eXtreme Gradient Boosting, SVM: Support Vector Machine, DT: Decision Tree, GNB: Gaussian Naive Bayes, KNN: K-Nearest Neighbors, AUC: Area Under the Curve.

Methods	Accuracy	Specificity	Recall	Precision	AUC
XGB	93.59	93.48	93.70	92.93	0.93
SVM	85.42	85.32	85.52	84.81	0.85
DT	83.60	83.50	83.70	83.00	0.83
GNB	79.93	79.84	80.02	79.36	0.80
KNN	87.69	87.59	87.80	87.07	0.87

**Table 2:** Summary of average results and standard deviations of DYI, MCC, F1 score, and kappa obtained from different machine learning models in the study. XGB: eXtreme Gradient Boosting, SVM: Support Vector Machine, DT: Decision Tree, GNB: Gaussian Naive Bayes, KNN: K-Nearest Neighbors, DYI: Dependent Youden Index, MCC: Matthews Correlation Coefficient.

Methods	DYI	MCC	F1 Score	Kappa
XGB	93.52	83.05	93.31	83.85
SVM	85.41	75.79	85.16	75.94
DT	83.60	74.18	83.35	74.43
GNB	79.93	70.92	79.69	71.06
KNN	87.69	77.81	87.43	77.96

## Data Availability

The datasets used and/or analyzed during the present study are available from the corresponding author on reasonable request. The data are not available to the public due to lack of patient authorization.
